# The Diseases of Infancy and Childhood

**Published:** 1897-04

**Authors:** 


THE DISEASES OF INFANCY AND CHILDHOOD. By L. EMMETT HOLT, A. M., M. D., Professor of Diseases of Children in the New York Polyclinic ; Attending Physician to the Babies' Hospital and to the Nursery and Child's Hospital, New York ; Consulting Physician to the New York Infant Asylum and to the Hospital for Ruptured and Crippled. Illustrated with nineteen full-page colored photogravure plates and 185 cuts inserted with the text. New York : D. Appleton & Co., Publishers, 72 Fifth avenue.

Part I. of this work includes chapters on the hygiene and general care of infants and young children, the growth and development of the body and a chapter devoted chiefly to the description of various therapeutic measures, such as stomach-washing, lavage, nasal-syringing, and the like. This chapter is so written and illustrated that various measures are made plain to those who have never had the opportunity of seeing them done.

Part II. comprises ten sections. Section one deals with diseases of the newly-born ; section two is entitled Nutrition. Nearly 100 pages are devoted to this subject, and the section is one of great value. Scorbutus and rickets appear here under the head of Diseases due to faulty nutrition.

Diseases of the digestive system properly follow the section on nutrition and naturally occupy a large space. The section devoted to diseases of the respiratory apparatus is of special excellence. Diseases of the circulatory system, of the nervous system, of the blood, lymph nodes and bones fill up the volume to section nine, which is devoted to the specific infectious diseases. Rheumatism and diabetes mellitus occupy the concluding chapters of the book.

It is difficult to say too much in praise of this work. The style is clear and concise ; the illustrations new and to the point ; the therapeutics rational and simple. The author's large experience has enabled him to produce a book which is, for the most part, original. Representing the experience of one of the foremost teachers of pediatrics in America today, the volume should find its way to many a physician's library. M. J. F.

